# 1715. In Vitro Antibacterial Activity of Cefiderocol against Difficult-to-treat Resistant (DTR) Gram-negative Pathogens in United States from SENTRY Antimicrobial Surveillance Program in 2020/2021

**DOI:** 10.1093/ofid/ofac492.1345

**Published:** 2022-12-15

**Authors:** Yoshinori Yamano, Miki Takemura, Dee Shortridge, Christine M Slover, Christopher M Longshaw, Roger Echols, Roger Echols, Roger Echols

**Affiliations:** Shionogi & Co., Ltd., Toyonaka, Osaka, Japan; Shionogi & Co., Ltd, Toyonaka, Osaka, Japan; JMI Laboratories, North Liberty, Iowa; Shionogi Inc., Florham Park, New Jersey; Shionogi B.V., London, England, United Kingdom; Infectious Disease Drug Development Consulting, Easton, Connecticut; Infectious Disease Drug Development Consulting, Easton, Connecticut; Infectious Disease Drug Development Consulting, Easton, Connecticut

## Abstract

**Background:**

Difficult-to-treat resistant (DTR) isolates are defined as resistance to all first-line high-efficacy, low-toxicity antibiotics (penicillins, cephalosporins, carbapenems and quinolones), leaving physicians with limited treatment options. Cefiderocol (CFDC) is a siderophore cephalosporin with activity against a wide variety of Gram-negative bacteria, including carbapenem-resistant Enterobacterales and non-fermenters. We evaluated the *in vitro* activity of cefiderocol against DTR isolates collected in United States as part of SENTRY surveillance program in 2020 and 2021.

In vitro susceptibility of cefiderocol and comparator agents to DTR isolates

**Figure:**
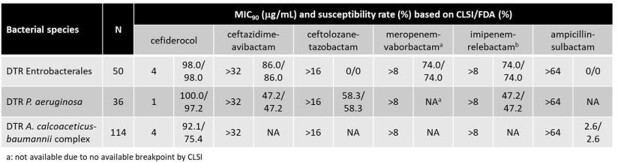

**Methods:**

Susceptibility testing was performed by broth microdilution according to CLSI guidelines. All antibiotics were tested in cation-adjusted Mueller-Hinton broth (CAMHB) except for CFDC, for which iron-depleted CAMHB was used. Susceptibility rate (%) was determined according to CLSI breakpoints, and DTR pathogens were defined as being resistant to cefepime, ceftazidime ceftriaxone (only for Enterobacterales), imipenem, meropenem, ciprofloxacin and levofloxacin according to CLSI/FDA breakpoints.

**Results:**

Among a total of 8,328 Enterobacterales, 2,241 *Pseudomonas aeruginosa*, and 586 *Acinetobacter calcoaceticus-baumannii* complex (ACB) clinical isolates from the United States, 50 Enterobacterales (0.6%), 36 *P. aeruginosa* (1.6%) and 114 ACB (19.5%) isolates showed a DTR phenotype, respectively. CFDC demonstrated its potent *in vitro* activity against these DTR isolates with MIC_90_ of ≤4 μg/mL with a susceptibility rate of ≥92% except for DTR ACB based on FDA breakpoint (75.4%). In contrast, MIC_90_s of β-lactam/β-lactamase inhibitor combination drugs showed lower activity (Table).

**Conclusion:**

Cefiderocol demonstrated potent *in vitro* activity against DTR isolates of Enterobacterales, *P. aeruginosa* and ACB, indicating cefiderocol has high potential for treating infections caused by these difficult-to-treat strains.

**Disclosures:**

**Yoshinori Yamano, PhD**, Shionogi: Employee **Miki Takemura, n/a**, Shionogi: Employee **Dee Shortridge, PhD**, AbbVie: Grant/Research Support|JMI Laboratory: Employee|Melinta: Grant/Research Support|Menarini: Grant/Research Support|Shionogi: Grant/Research Support **Christine M. Slover, PharmD**, Shionogi: Employee **Christopher M. Longshaw, PhD**, Shionogi: Employee **Roger Echols, MD**, Shionogi: Advisor/Consultant **Roger Echols, MD**, Shionogi: Advisor/Consultant **Roger Echols, MD**, Shionogi: Advisor/Consultant.

